# The association between the time of alcohol drinking and injury risk in Thailand: a cross‐sectional emergency department study

**DOI:** 10.1186/s13011-021-00365-y

**Published:** 2021-03-30

**Authors:** Bundit Sornpaisarn, Sarnti Sornpaisarn, Jürgen Rehm

**Affiliations:** 1 Institute for Mental Health Policy Research, Centre for Addiction and Mental Health, 33 Russell Street, Ontario M5S 2S1 Toronto, Canada; 2 Dalla Lana School of Public Health, University of Toronto, 155 College Street, 6th floor, M5T 3M7 Toronto, Ontario Canada; 3 Faculty of Public Health, Mahidol University, 420/1 Ratchawithi Road, Thung Phaya Thai, Ratchathewi, 10400 Bangkok, Thailand; 4 Faculty of Health Science, McMaster University, 1280 Main Street West, L8S 4L8 Hamilton, Ontario Canada; 5 World Health Organization / Pan American Health Organization Collaborating Centre, Centre for Addiction and Mental Health, 33 Ursula Franklin Street, M5S 2S1 Toronto, Ontario Canada; 6 Campbell Family Mental Health Research Institute, Centre for Addiction and Mental Health, 33 Ursula Franklin Street, M5S 2S1 Toronto, Ontario Canada; 7 Department of Psychiatry, University of Toronto, 250 College Street, 8th floor, M5T 1R8 Toronto, Ontario Canada; 8 Centre for Interdisciplinary Addiction Research, University of Hamburg, Martinstraße 52, 20246 Hamburg, Germany; 9 Institute of Clinical Psychology and Psychotherapy & Center of Clinical Epidemiology and Longitudinal Studies (CELOS), Technische Universität Dresden, Chemnitzer Str. 46, 01187 Dresden, Germany; 10 Department of International Health Projects, Institute for Leadership and Health Management, I.M. Sechenov First Moscow State Medical University, Trubetskaya str., 8, b. 2, 119992 Moscow, Russian Federation

**Keywords:** Alcohol, Emergency department, Injury, Drinking behaviour, Thailand, Time

## Abstract

**Background:**

Although the relationship between acute alcohol consumption and injuries is well recognized, studies exploring how the time of day the drinking commences affects alcohol-related injuries have been scarce. This contribution examines the associations between the time at which the drinking began and the duration of the drinking, the volume of alcohol consumed, the injury type, and the blood alcohol concentration (BAC) level.

**Method:**

This study employed a cross-sectional survey, which was conducted in two hospital emergency departments (ED) in Chiangmai Province, Thailand. The sample was composed of 519 injured patients aged 18 years and older. Outcome measures included the BAC and type of injury. Exposures included the quantity of alcohol consumed, the time the drinking commenced, and the pattern of drinking involved.

**Results:**

The injured patients who drank alcohol within six hours prior to sustaining their injury were more likely to get injured and present themselves at the ED at night (20:00–04:00) compared to those who sustained an injury but did not drink in the hours prior. However, this relationship was only true for unintentional injuries, not intentional ones. The majority of participants consumed their first drink between 16:00 and 20:00. On average, among the 104 patients who drank prior to sustaining an injury, the total amount of alcohol consumed was 6.9 drinks, the duration of drinking was 2.6 h, the rate of drinking was 6.0 drinks/hour, and the BAC was 0.119 gm%. Every drink increased the BAC by 0.012 gm% and each year of increasing age increased the BAC by 0.003 gm%. People who were older, less educated, and drank more frequently tended to have their first drink earlier than other drinkers. An earlier start to their drinking resulted in a faster pace of drinking and a higher BAC.

**Conclusions:**

BAC increased with the total amount of alcohol consumed and the age of the drinker. Different groups of people had their first drink at different times of the day, resulting in differences in the rate of drinking, the BAC, the time of injury, and the time they presented to the ED after injury.

## Introduction

Injuries can be caused in many ways, including, but not limited to, violence, self-harm, road traffic collisions, burns, drownings, and falls. It can have an immeasurable impact on individuals’ lives and those around them, causing morbidity and financial costs. Injuries were the second leading cause of direct medical costs in Canada [1] and in the U.S. [2]. On a global scale, injuries cause significant burden. According to the Institute for Health Metrics and Evaluation, injuries contributed to over 4 million deaths and 252 million Disability-Adjusted Life Years (DALYs) worldwide in 2017 [3]. Injury-related deaths and DALYs were also high in Thailand. In 2017, injuries contributed to 48,268 deaths and 2.48 million DALYs in Thailand, accounting for 10 and 13 % of the nation’s total deaths and DALYs, respectively [3]. Road injuries ranked first in the disease and injury category that caused the most premature deaths in Thailand in 2017 [3].

Alcohol consumption, especially heavy drinking, is a clear factor for many chronic health problems and injuries, and creates a great burden to society [4, 5]. Many acute injuries can also be attributable to alcohol use; among injured patients who end up in the emergency department (ED), alcohol is more likely to be present than in non-injured ED patients [6]. The proportion of injured patients with alcohol exposure has been found to range from 5.9 % in Ontario, Canada to 41.7 % in Auckland, New Zealand [7], and up to 60 % in Cape Town, South Africa [8]. There have been many ED studies which examined the impacts of alcohol consumption in increasing the risk for injury. These studies found that acute alcohol consumption, both alcohol exposure and the quantity of alcohol consumed, increased the risk of injury for patients seeking health services in an ED [6, 9–15]; however, the effects varied depending on gender and cause of injury (i.e., traffic, fall, violence) [7, 14–18]. Alcohol consumption is the largest contributor to the global burden of injuries [19]. We previously conducted a case-crossover study using ED settings in Chiang-Mai, Thailand in 2016 [20] and found that the prevalence of alcohol-related injuries was 19.5 %, and that alcohol consumption increased the odds of a patient’s injury five-fold (Odds Ratio (OR) = 5.0; 95 % Confidence Interval (CI): 3.0, 8.2) compared to patients who did not drink. Furthermore, each additional drink consumed increased the risk (odds) of being injured by 30 %. Our study yielded similar results to studies from other countries (see studies mentioned above).

Although there have been many studies exploring the relationships between alcohol exposure, the amount of alcohol consumed, and the risk of subsequent injuries, as described above, only a small number of studies examined the effects of other factors. Only a handful of studies assessed the effects of the usual drinking patterns (i.e., drinking every day or every week) [21, 22], the drinking context (i.e., drinking in public or private) [23, 24]), and the beverage type consumed (i.e., beer or spirits) [25]. Currently, there are no studies in the literature which explore the effects of the time of day that alcohol consumption began prior to the injury event, or the risk of being injured, using an ED setting. This study aims to explore associations between the time of day of onset of drinking, duration of drinking, rate of drinking, and various drinking outcomes, such as injury type, and blood alcohol concentration (BAC).

## Methods

### Design, sample and data collection

Study design and data collection tools were developed by the World Health Organization (WHO) collaborative study on alcohol and injuries [9]. The present study was conducted in the EDs of two major hospitals in Chiang-Mai, Thailand: Maharaj Hospital, a university teaching hospital with roughly 1,400 hospital beds, and Nakornping General Hospital, with approximately 600 hospital beds. Ethics approval was granted by the ethics committee of Chiang Mai University, Thailand, on April 19, 2016 (Ethics Approval #11/2016).

Data was collected from patients aged 18 years or older who were admitted to a hospital ED within 6 h of an injury between June and August of 2016, for a total of 519 participants (response rate of 89 %). Every injured patient recorded during the night (0:00–8:00) and morning (8:00–16:00) shifts, and every other injured patient during the evening (16:00–0:00) shift was approached and asked to participate in the study. To correct for different probabilities of sampling and to be representative for ED visits as a whole, weights were introduced, where possible. However, Fisher’s exact test could not be conducted as weight, and thus we report a Chi2 for weighted and Fisher’s exact test for unweighted counts. Trained field staff collected data from study samples using the WHO / National Institute on Alcohol Abuse and Alcoholism (NIAAA) Alcohol & Injury structured interview schedule. The BAC of each participant was obtained using a breathalyzer administered immediately upon the patient’s arrival at the ED. See [20] for details. Our study used four brand new breathalyzers (the Alco-Sensor III Breathalyzer model, the Alco-Sensor FST brand). This breathalyzer model provides breath alcohol measurement results equivalent to BAC.

### Measures

We were interested in five sets of variables. (1) The demographic variables consisted of sex, age, education, and place of residence. (2) The time-related alcohol consumption behaviours and their consequences, including the weekday and the time of the first drink, the weekday and time of the last drink, the time of injury, and the time patients presented at the ED. The WHO questionnaire that we used had questions pertaining to the day and time of the first drink, the last drink, the patient recruitment time (the time spent in the ED), the period of time between the last drink and the injury, and the period of time between the injury and arrival at the ED. We constructed the time of the injury and the categorical variables based on the day and time of the first and last drinks. It should be noted—especially for researchers who would like to analyze time-related alcohol consumption behaviour—that the period of time that bridges midnight, and therefore encompasses two different dates when constructing the duration variable, must be taken into account. (3) The variables for alcohol consumption within six hours prior to the injury consist of total alcohol consumption, duration of alcohol consumption, and number of drinks consumed per hour. In this study, alcohol consumption and the amount of alcohol intake for each participant in the 6-hour period prior to the injury were recorded. The volume of alcohol consumed was standardized by converting the number and volume of alcoholic beverages consumed into ml of pure ethanol. A standard drink was assumed to contain 16 ml of ethanol. We constructed a variable for duration of drinking by subtracting the time of the last drink by the time of the first drink, taking into account the dates of both drinking times. The rate-of-drinking variable was constructed by dividing the amount of drinking by the duration of drinking. (4) The usual drinking patterns of the participants are also recorded. (5) Our drinking outcome variables of interest included BAC, type and mode of injury. The types of injury included unintentional injury, intentional injury inflicted to oneself, and intentional injury inflicted by someone else. The modes of injury covered injuries related to vehicle crashes (including driver, passenger, and pedestrian); blunt force injury; stab, cut, bite; fall, trip; and others (covering sexual assault, choking, hanging, poisoning, burning with fire, flame, heat, or hot liquid, and others). All injury categories that had less then 30 recorded incidents were included in the ‘others’ category. Please note that the types and modes of injuries were derived from the answers patients provided to questions regarding why and how they were injured, respectively. These two questions are independent of each other. No patient answered ‘unknown’ to both questions.

### Statistical analysis

Differential sampling probabilities between nurse shifts were adjusted by weights. The patients who arrived during the night (0:00–8:00) and morning (8:00–16:00) shifts were thus assigned half the weight of the patients from the evening shift (16:00–0:00). Our study calculated percentages and means with 95 % Confidence Intervals (CI) to describe the distribution of the categorical and continuous variables, respectively. We employed the tabulation with the chi-square test to explore the relationships between the categorical variables. For cases where Fisher’s exact test was indicated, we used unweighted counts, but report the weighted chi-square tests as well. To examine the relationship between the continuous variables, we calculated means and 95 % CIs for each of the time periods for the first drink and compared them using an ANOVA technique. Lastly, multiple linear regression analyses were utilized to examine which of the 13 factors were likely to influence the BAC. We started by analysing the binary relationships between each of the 13 factors and then sequentially added sets of potential determinants to the regression model. All statistical tests applied a p-value of 0.05 as a statistically significant level. We used STATA software 15.0 for all analyses.

The analysis was not pre-registered and the results should be considered exploratory.

## Results

Of the 519 injured patients who participated in the study, the majority were male (61 %), 18–44 years of age (67 %), had 7 or more years of education (70 %), and lived in the city (78 %). The prevalence of alcohol exposure during the 6 h prior to sustaining the injury/injuries was 19.5 % (95 % CI: 15.9 %, 23.1 %) (see details in [20]). The following data are not shown in the tables provided: the BACs for patients who reported drinking 1–2, 3–4, 5–6, and 7 + drinks during the 6 h prior to being injured were 0.020, 0.0990, 0.9993, and 0.144 gm%, respectively (*p*-value = 0.002). The BACs were 0.106, 0.144, and 0.153 gm% for patients who drank ≤ 5, > 5 and ≤ 10, and > 10 drinks per hour, respectively (*p*-value = 0.123). Two patients reported not drinking during the six hours prior to being injured but were found to have BAC readings of 0.030 and 0.180 gm%.

Table 1 provides a summary of the variation in injury time among 519 injured patients and their status of alcohol exposure within the six hours before they sustained their injury. Those who drank within six hours prior to their injuries (101 patients, 20 %) were more likely to be injured and visit the ED between 20:00 and 04:00 (69.6 and 81.1 %, respectively). Among those who did not drink, 85.0 and 89.8 % were injured and visited the ED between 08:00 and 20:00, respectively. 82.9 % of those who drank within the six-hour period prior to sustaining their injuries were unintentionally injured; 67.8 % of them visited the ED from 20:00 to 04:00. In contrast, 95.7 % of those who did not drink prior to their injury were unintentionally injured and 74.8 % of them visited the ED from 08:00 to 20:00. In term of mode of injury, injured patients were injured by traffic accidents, stabs/cuts/bites, or falls/trips, respectively. Regardless of mode of injury, the majority of patients who drank alcohol in the six hours prior to sustaining their injuries visited the ED between 20:00 and 04:00, while the majority of patients who did not drink visited the ED between 8:00 and 20:00.

**Table 1:** Percentage of injured patients with the time of injury and the arrival time at the ED, stratified by the status of alcohol drinking within the six hours prior to the injury and type of injury

		Drink	N			Time				Statistic
		alcohol^a^	(519)	8-12	12-16	16-20	20-24	00-04	04-08	*p*-value
				(Row %)	(Row %)	(Row %)	(Row %)	(Row %)	(Row %)	
Time of injury ^b^		Yes	101	6.7	8.9	9.6	44.4	25.2	5.2	Chi 2 = 158.9
		No	418	19.6	20.5	32.4	12.5	4.3	10.7	*p*<0.001***
Time arriving at the ED^b^		Yes	101	5.2	5.2	8.9	47.0	34.1	9.6	Chi 2 = 143.3
		No	418	20.5	16.9	32.8	19.6	5.9	4.3	*p*<0.001***
Time arriving at the ED, by types of injury ^c^	Unintentional	Yes	87	8.1	5.8	5.8	27.6	40.2	12.6	Chi 2 = 112.3
		No	396	28.8	23.0	23.0	13.3	6.6	5.6	Fisher’s exact *p*<0.001***
	Intentional,	Yes	2	0.0	0.0	0.0	0.0	50.0	50.0	Chi 2 = 1.5
	self-inflicted	No	4	0.0	50.0	0.0	0.0	25.0	25.0	Fisher’s exact *p*=1.000
	Intentional,	Yes	16	0.0	12.5	6.3	6.3	68.8	6.3	Chi 2 = 3.4
	by someone else	No	14	7.1	14.3	7.1	21.4	42.9	7.1	Fisher’s exact
										*p*=0.742
	Traffic	Yes	66	6.1	7.6	6.1	242	40.9	15.2	Chi 2 = 46.7
	accident	No	149	28.9	20.1	18.1	13.4	10.1	9.4	Fisher’s exact
Time arriving at the ED, by mode of injury ^c^										*p*<0.001***
	Blunt force	Yes	10	0.0	0.0	0.0	10.0	70.0	20.0	Chi 2 = 16.2
	injury	No	27	22.2	22.2	18.5	14.8	11.1	11.1	Fisher’s exact
										*p*=0.004**
	Stab, cut,	Yes	11	9.1	9.1	9.1	36.4	36.4	0.0	Chi 2 = 21.9
	bite	No	131	32.1	26.0	25.2	9.2	6.1	1.5	Fisher’s exact
										*p*<0.002**
	Fall, trip	Yes	11	18.2	0.0	0.0	27.3	45.5	9.1	Chi 2 = 22.5
		No	78	26.9	19.2	29.5	14.1	5.1	5.1	Fisher’s exact
										*p*<0.001***
	Others	Yes	7	0.0	14.3	14.3	14.3	57.1	0.0	Chi 2 = 8.5
		No	29	10.3	34.5	13.8	27.6	10.3	3.5	Fisher’s exact
										*p*=0.182

* *p*<0.05, ** *p*<0.01, *** *p*<0.001

^a^ Drank alcohol within six hours prior to the injury

^b^ Weighted samples

^c^ Unweighted analyses

Table 2 shows the distribution of the time of the first drink stratified by sex, age, education, and place of residence among 104 injured patients who drank within six hours prior to the injury. 81.8 % of young adult patients (aged 18–24 years old) started their first drink between 20:00 and 24:00 while older people tended to start drinking earlier with advancing age (*p*-value < 0.001). The majority (80.8 %) of patients with 7 + years of education had their first drink between 16:00 and 24:00, whereas those with lower education levels had their first drink earlier in the day (*p*-value = 0.001). Similarly, the majority of patients had their first drink between 16:00 and 24:00; however, frequent drinkers had their first drink earlier. Those who had their first drink between 08:00 and 12:00 were drinkers who drank every day or almost every day, while those who had their first drink from 12:00 to 16:00 consisted of drinkers who drank at least once a week (data not shown in Table 2).

**Table 2:** Percentage of injured patients who drank alcohol within six hours prior to the injury with the time of first drink, stratified by sex, age, education, and residence

		Time of first drink						Statistic
	N = 104^a^	8–12	12–16	16–20	20–24	00–04	04–08	(*p*-value)
	(Col %)	(Row %)	(Row %)	(Row %)	(Row %)	(Row %)	(Row %)	
Sex								
Male	78.1	3.7	9.8	23.2	53.7	8.5	1.2	Chi 2 = 7.4
Female	21.9	0.0	4.6	9.1	68.2	9.1	9.1	Fisher’s exact
								*p* = 0.236
Age								
18–24	41.9	0.0	4.6	4.6	81.8	9.1	0.0	Chi 2 = 56.6
25–44	43.8	4.4	6.7	31.1	46.7	11.1	0.0	Fisher’s exact
45–59	11.4	8.3	33.3	25.0	16.7	0.0	16.7	*p* < 0.001***
60+	2.9	0.0	0.0	66.7	0.0	0.0	33.3	
Education								
0	8.6	22.2	11.1	22.2	33.3	11.1	0.0	Chi 2 = 33.3
1–6	16.2	0.0	17.7	41.2	29.4	0.0	11.8	Fisher’s exact
7–12	46.7	0.0	6.1	16.3	65.3	12.2	0.0	*p* = 0.008**
13+	28.6	3.5	6.9	13.8	65.5	6.9	3.5	
Resident								
Suburb	38.1	5.0	15.0	15.0	52.5	10.0	2.5	Chi 2 = 5.3
City	61.9	1.6	4.7	23.4	59.4	7.8	3.1	Fisher’s exact
								*p* = 0.364
Usual drinking patterns								
Every day	14.3	6.7	33.3	26.7	13.3	6.7	13.3	Chi 2 = 39.0
Almost every day	22.9	8.3	4.2	25.0	54.2	8.3	0.0	Fisher’s exact
3–4 times a week	18.1	0.0	10.5	10.5	68.4	5.3	5.3	*p* = 0.036*
1–2 times a week	19.1	0.0	5.0	15.0	65.0	15.0	0.0	
2–3 times a month	12.4	0.0	0.0	30.8	69.2	0.0	0.0	
Once a month or less	13.3	0.0	0.0	15.4	69.2	15.4	0.0	

* *p* < 0.05, ** *p *< 0.01, *** *p* < 0.001

^a^ Unweighted samples

Table 3 illustrates that 79.1 % of injured patients consumed their fist drink during 16:00 to 24:00, whereas 72.8 %, 70.7 %, and 71.3 % finished their last drink, were injured, and presented themselves to the ED between 20:00 and 04:00. It also shows the averages for the duration of drinking, the duration between the last drink and the injury, and the duration between the injury and the time they arrived at the ED, stratified by the time of the first drink. The average times were 2.6, 0.9, and 1.4 h for these three time periods, respectively. There was no statistically significant difference between the time of the first drink with respect to each of these three durations. As a result, the duration between the first drink and the injury was 3.5 (3.0–4.1) hours and the duration from the first drink to presentation at the ED was 6.3 (5.5–7.1) hours.

**Table 3:** Time of first drink, last drink, injury, and at the Emergency Department as well as the duration between each event

	Time of first drink^a^		Time of last drink^a^		Time of the injury^a^			Time at the Emergency Department^a^
	Distribution by time	Duration of drinking	Distribution by time	Duration between the last drink and the time of injury	Distribution by time		Duration between the time of injury and the time at the emergency department	Distribution by time
*N* = 104	(Col %)	Mean (95 % CI)	(Col %)	Mean (95 % CI)	(Col %)		Mean (95 % CI)	(Col %)
08–12	2.2	2.2 (-0.4–4.9)	5.2	1.2 (0.2–2.1)	6.0		1.1 (0.8–1.4)	5.2
12–16	9.7	2.1 (-0.1–4.4)	9.6	0.4 (0.2–0.6)	9.0		1.0 (1.0–1.0)	5.2
16–20	25.4	3.3 (2.3–4.4)	10.3	0.9 (-0.1–1.9)	9.8		1.6 (0.4–2.8)	8.8
20–24	53.7	2.6 (2.0–3.3)	47.1	0.8 (0.5–1.1)	44.4		1.4 (1.1–1.7)	36.8
00–04	6.7	1.2 (0.5–1.8)	25.7	1.0 (0.6–1.5)	26.3		1.5 (1.2–1.9)	34.5
04–08	2.2	0.8 (-1.0–2.7)	2.2	1.8 (-2.7–6.2)	4.5		1.7 (0.8–2.5)	9.6
		*F* = 1.34 *P* = 0.252		*F* = 1.00 *P* = 0.402			F = 0.79 P = 0.562	
Average		2.6 (2.1–3.1)		0.9 (0.7–1.1)			1.4 (1.2–1.6)	

* *p* < 0.05, ** *p* < 0.01, *** *p* < 0.001

^a^ Weighted sample

Table 4 provides the data on the drinking outcomes, stratified by the time of the first drink. On average, the total number of drinks consumed was 6.9 drinks, the duration of drinking was 2.6 h, and the drinking rate was 6.0 drinks/hour. The average BAC among the injured patients who drank within six hours prior to their injury was 0.119 gm%. The number of drinks consumed per hour and the BAC level were significantly heterogeneous regarding the time of first drink, with a *p*-value of < 0.001 and 0.003, respectively. Those who drank at a faster rate of 11.4 drinks/hour were patients who had their first drink between 12:00 and 16:00. Those people who had the highest BAC scores (0.263 gm% average) were those who had their first drink between 08:00 and 12:00 and consumed about 9.7 drinks. These morning drinkers were all male, with an average age of 42.3 years old, and had an average 7 years of education (data not shown).

**Table 4:** Time of first drink and drinking outcomes: total number of drinks, duration of drinking session, number of drinks per hour, and BAC

			Total number of drinks		Duration of drinking session		Number of drinks per hour		BAC	
Time of first drink	*N*^a^ = 104 (%)		(Drink)		(Hour)		(Drink / hour)		(gm%)	
Average			6.9 (6.4–7.5)		2.6 (2.1–3.1)		6.0 (3.8–8.3)		0.119 (0.101–0.137)	
08–12	2.2		9.7 (8.2–11.1)		2.2 (-0.4–4.9)		5.4 (-2.5–13.2)		0.263 (0.249–0.278)	
12–16	9.6		7.0 (4.5–9.5)		2.1 (-0.1–4.4)		11.4 (1.9–20.9)		0.174 (0.104–0.244)	
16–20	25.2		7.1 (6.1–8.1)		3.3 (2.3–4.4)		3.0 (2.2–3.9)		0.144 (0.103–0.185)	
20–24	54.1		6.9 (6.1–7.7)		2.6 (2.0–3.3)		4.6 (3.6–5.6)		0.092 (0.075–0.110)	
00–04	6.7		6.4 (3.7–9.2)		1.2 (0.5–1.8)		9.6 (0.3–18.8)		0.111 (-0.001–0.224)	
04–08	2.2		4.7 (-4.7–14.1)		0.8 (-1.0–2.7)		43.7 (-120.8–208.1)		0.083 (-0.275–0.442)	
			*F* = 0.73*P* = 0.604		*F* = 1.33*P*=0.257		*F* = 7.86*P* < 0.001***		*F* = 3.9*P* = 0.003**	

* *p* < 0.05, ** *p* < 0.01, *** *p* < 0.001

^a^ Weighted samples

Table 5 illustrates the data on the injury outcomes, stratified by the time the first drink was consumed. The most prevalent type of injury was unintentional (83.7 %) and the most prevalent mode of injury was traffic accident. The time of the first drink was not related to the type or mode of injury.

**Table 5:** Time of first drink and type and mode of injury

		Type of injury			Mode of injury				
		Unintentional	Intentional, self-inflicted	Intentional, by someone else	Traffic accident	Blunt force injury	Stab, cut, bite	Fall, trip	Others
Time of first drink	N^a^ (104)	(Row %)	(Row %)	(Row %)	(Row %)	(Row %)	(Row %)	(Row %)	(Row %)
Average		83.7	1.9	14.4	63.5	9.6	10.6	10.6	5.8
08–12	3	33.3	0.0	66.7	33.3	0.0	33.3	0.0	33.3
12–16	9	88.9	0.0	11.1	88.9	0.0	11.1	0.0	0.0
16–20	21	85.7	4.8	9.5	61.9	9.5	4.8	14.3	9.5
20–24	59	83.1	1.7	15.3	59.3	11.9	11.9	11.9	5.1
00–04	9	88.9	0.0	11.1	88.9	11.1	0.0	0.0	0.0
04–08	3	100.0	0.0	0.0	33.3	0.0	33.3	33.3	0.0
		Chi 2 = 9.058, Fisher’s exact *P* = 0.481			Chi 2 = 19.068, Fisher’s exact *P* = 0.552				

* *p* < 0.05, ** *p* < 0.01, *** *p* < 0.001

^a^ Unweighted samples

Table 6 demonstrates the regression coefficients in the simple linear regression models of the factors potentially predicting the BAC level. In the simple bivariate analysis, increasing age and total number of drinks were statistically significant in increasing BAC, while drinking the first drink from 16:00 to 24:00, finishing the last drink from 20:00 to 04:00, and the more frequent drinking patterns were statistically significant in reducing BAC levels compared to those who started their first drink during other periods. However, these findings were not consistent with the multiple regression analysis. The multiple linear regression concluded that among injured participants, the total number of drinks was correlated to BAC, with each drink increasing BAC by 0.012 gm% per drink (95 % CI: 0.006–0.019). Age also had a significant correlation to BAC, increasing it by 0.003 gm% per year of age (95 % CI: 0.001–0.005).

**Table 6:** Regression coefficients of the factors predicting BAC: demographic variables, alcohol drinking variables, time of initiation of alcohol drinking, and the usual drinking patterns

	Bivariate	Multivariate			
	Co-efficient (*p*-value)	Model 1: demographic data	Model 2: model 1 + drinking 6 h prior to the injury	Model 3: model 2 + the time of drinking	Model 4: model 3 + the usual drinking habits
1.Sex	-0.041 (0.094)	-0.032 (0.185)	-0.011 (0.614)	-0.015 (0.516)	-0.013 (0.587)
2.Age	0.003 (0.000)***	0.003 (0.000)**	0.003 (0.000)***	0.003 (0.000)***	0.003 (0.000)***
3.Education	-0.016 (0.108)	0.005 (0.625)	0.008 (0.409)	0.010 (0.282)	0.011 (0.260)
4.Resident	-0.014 (0.475)	-0.001 (0.951)	0.009 (0.573)	0.012 (0.494)	0.012 (0.472)
5.Total number of drinks	0.013 (0.000)***		0.013 (0.000)***	0.013 (0.000)***	0.012 (0.000)***
6.Drinking duration	0.002 (0.679)		0.002 (0.641)	0.001 (0.795)	0.001 (0.775)
7.Number of drinks per hour	0.002 (0.218)		0.002 (0.266)	0.001 (0.726)	0.001 (0.638)
8.Time between the last drink and the ED	-0.007 (0.069)		-0.004 (0.206)	-0.004 (0.207)	-0.004 (0.202)
9.Drinking the first drink during the popular period	-0.044 (0.042)*			-0.030 (0.189)	-0.028 (0.228)
10.Drinking the first drink during the early part of the week	0.002 (0.650)			-0.010 (0.803)	-0.013 (0.742)
11.Drinking the last drink during the popular period	-0.051 (0.010)*			-0.010 (0.626)	-0.007 (0.734)
12.Drinking the last drink during the early part of the week	0.002 (0.660)			0.013 (0.740)	0.017 (0.674)
13.Drinking frequently usually	-0.014 (0.004)**				-0.003 (0.540)
P-value of the model		0.0017**	0.0000***	0.0000***	0.0000***
R-squared		0.1711	0.3841	0.4017	0.4045

* *p* < 0.05, ** *p* < 0.01, *** *p* < 0.001

## Discussion

This study explored the association between the time of day when drinking was initiated and the outcomes for patients who drank alcohol within six hours prior to sustaining an injury and presenting to an emergency department. We found that study participants who had their first drink at any time during the day were, on average, at the greatest risk of injury about 3.5 h later, and likely to attend the ED 6.3 h after their first drink. However, the patients who drank before sustaining an injury were more likely to present themselves at the ED at night (20:00–04:00) compared to those who did not drink before their injury, regardless of the mode of injury. This relationship was true only for the unintentional injuries. An explanation is that the majority of our study participants (79.1 %) drank from 16:00 to 24:00 with an average duration of drinking of 2.6 h. The average duration between the last drink and the injury was 0.9 h, and 1.4 h elapsed between the time of injury and their presentation at the ED. These average times combined resulted in most study participants getting to the ED at the above-mentioned time, between 20:00 and 04:00. This finding reaffirms the reasoning behind why a night-time car crash is often used as a proxy indicator for an alcohol-related accident [26].

On average, study participants drank 6.9 drinks at a rate of 6.0 drinks/hour before being injured. We found that those who started drinking between 12:00 and 16:00 did so at the highest drinking rate of 11.4 drinks/hour. This may be due to drinkers being in a rush to drink quickly over their lunch period. Our study also observed that the patients who started their first drink between 08:00 and 12:00 tended to be male, older, and have less education. They were likely to be more problematic drinkers: they drank frequently, drank excessively, and had high BAC values. Drinking every day or almost every day is not common in Thai culture. Only 9.5 % of males and 3.7 of females drink every day or almost every day [27]. Only 5.9 % of males and 0.4 % of females are heavy drinkers in Thailand [28]. Bellis et al. (2010) found that excessive drinkers tended to drink greater amounts, for longer periods, and drank faster, resulting in higher BAC levels compared to non-excessive drinkers [29]. Assanangkornchai et al. (2010) found that a greater number of older Thai drinkers (25–44 and 45–65 years old) drank every day or almost every day compared to their younger counterparts (12–19 and 20–24 years old) [27]. Jansirimongkol et al. (2011) found that Thai people diagnosed with alcohol dependence were more likely to be male, aged 25–44 years old, with less than 12 years of education, and single [30].

The finding that the total number of drinks increased the BAC level is similar to the findings of many of the other studies mentioned above. Interestingly, our study found that the BAC level increased with age after controlling for other factors, including the total number of drinks consumed. One explanation for this phenomenon is that the activity of the enzymes involved in ethanol metabolism (including acetaldehyde dehydrogenase and cytochrome P-4502E1) diminishes with age [31, 32]. Moreover, older people have poorer water distribution volume compared to younger people because lean body mass decreases and adipose tissue increases with age [33, 34]. These two factors lead to higher BAC levels among older drinkers [31]. Likewise, Perkin et al. (2001) found that young people aged 18–24 years old had a relatively lower BAC level relative to the legal limit in the US (0.08gm%), even after binge- drinking (5+/4 + drinks per occasion for males and females) [35].

Our study provides some interesting insights into the public health implications regarding the drinking behaviours of the patients who drink alcohol in the six hours prior to their sustaining their injuries. First, starting the first drink at any time of the day will increase the risk of injury uniformly. Second, most study participants had their first drink between 16:00 and 24:00, finished their last drink, sustained their injury, and then presented themselves to the ED between 20:00 and 04:00. Third, more frequent drinkers, older drinkers, and less educated drinkers seemed to have their first drink earlier in the day. Early-start drinkers seemed to be problematic drinkers who drank the fastest and/or the most. This relationship could be used to identify early drinking as a special risk factor. Fourth, increased age is associated with higher BAC levels. Lowering thresholds of the low-risk alcohol drinking guideline according to age, as done by some European countries, may be a good strategy to prevent alcohol-related harmful effects imposed on consumers.

To our knowledge, there are currently no ED studies exploring the effect of the start time of drinking on drinking outcomes. However, some limitations to this study must be mentioned. There are three limitations embedded in this study’s design: no information on the severity of the injury was available, samples were non-representative (they did not include those who did not seek treatment at the ED or who died before reaching the ED), and no information was available regarding other substances which may have been used concurrently (for details, see [20]). There are three more limitations specifically for this study which address the analysis of the time of drinking commencement. Firstly, recall bias for all event times may be present. The respondents had to recall several specific time-points. Calculations of the time of injury yielded inconsistent results when compared to calculations based on the time of the last drink, and calculations based on the time patients presented to the ED. Secondly, a greater sample size is needed to provide more power, since there are many variables to take into account. This kind of analysis using a larger sample size is needed to explore the complex relationship between the drinking start-times and outcomes. Thirdly, patients did not tell the truth about their drinking; however, there were only two patients who denied drinking during the six hours prior to being injured but were found to have positive BACs. Fortunately, our study found dose-response relationships between the BAC and the number of drinks consumed in the six hours prior to receiving the injury, indicating that our participants’ answers were reliable.

## Conclusions

Different groups of people seem to have their first drink at different times of the day, resulting in differences in the rates of drinking, BAC, the time of injury, and the time patients presented to the ED. To prevent alcohol-related unintentional injuries, government officials and relevant sectors should focus their efforts on intervening for alcohol drinking during evening and the early night-time hours since the majority of alcohol-related unintentionally injured patients drank alcohol from 16:00 to 24:00. More problematic drinkers who were older, less educated, and drank more frequently tended to have their first drink earlier in the day than their counterparts, and these groups may need a specific intervention, such as a brief intervention. BAC levels increased with the total amount of alcohol consumed and with the age of the drinker; hence, heavy drinking among older people might be of concern to public health personnel as well.

## Data Availability

The datasets used and/or analysed during the current study are available from the corresponding author on reasonable request.

## Abbreviations

ANOVA: Analysis of variance
BAC: Blood alcohol concentration
CI: Confidence interval
DALY: Disability-adjusted life year
ED: Emergency department
NIAAA: National Institute on Alcohol Abuse and Alcoholism
WHO: World Health Organization
